# Psychometric properties of the Spanish version of the Flourishing Scale in the Honduran population

**DOI:** 10.3389/fpsyg.2023.1223269

**Published:** 2023-07-20

**Authors:** Miguel Landa-Blanco, Antonio Cortés-Ramos, Gabriela Vásquez, Yarell Reyes, Yarani Echenique

**Affiliations:** ^1^Master’s Degree in Clinical Psychology, School of Psychological Sciences, National Autonomous University of Honduras, Tegucigalpa, Honduras; ^2^Department of Developmental Psychology and Education, Faculty of Psychology and Speech Therapy, University of Malaga, Malaga, Spain

**Keywords:** Flourishing, psychometric properties, factorial validity, reliability, convergent validity, divergent validity, measurement invariance

## Abstract

Studying Flourishing is important to understand wellbeing. The current study aimed to determine the psychometric properties of the Spanish version of the Flourishing Scale (FS) in the Honduran population. The primary sample consisted of 422 residents of the Central District of Honduras; this included 275 (65.17%) women and 147 men (34.83%). Their average age was 28.18 years (*SD* = 10.58). Findings from the Exploratory Factor Analysis and Confirmatory Factor Analysis support a unidimensional factor structure. The FS achieved a high internal consistency with McDonald’s *ω* = 0.89, 95% *CI* [0.86, 0.91]. The average inter-item correlation was 0.48, 95% *CI* [0.43, 0.53]. Using Student’s *t*-test for paired samples, results indicate that none of the FS items varied significantly between baseline and post-test. Additionally, Spearman’s rho was used to correlate test–retest scores; this yielded a statistically significant correlation coefficient of 0.66. The Flourishing Scale had adequate convergent validity with the Subjective Happiness Scale (*r* = 0.70) and the PANAS-Positive Affect Subscale (*r* = 0.70) (*p* < 0.001). In contrast, it correlates inversely with the PANAS-Negative Affect Subscale (*r* = −0.34) and the PHQ-9 (*r* = −0.51). Strict measurement invariance for sex was supported. The results indicate that the Flourishing Scale has robust psychometric properties for the Honduran population. Practical implications for public policy are discussed.

## 1. Introduction

According to the World Health Organization, health is a holistic state of physical, psychological, and social wellbeing, not merely the absence of disease (World Health Organization, 1946). This conceptualization transcends the reductionist pathology-based approach focused on the negative aspects of wellbeing. Focusing on both positive aspects of wellbeing (such as Flourishing), as well as negative facets, such as psychopathology, allows for a more complete understanding of an individual’s overall psychological health (Antaramian et al., 2010; García-Álvarez et al., 2020; Barragán and Ramsés, 2021). In this regard, the field of positive psychology is particularly important. Positive psychology focuses on the qualities and other facets that allow people to live a happy, productive life; to grow personally; and to develop the different capacities and skills that are considered important to promote wellbeing, for example, autonomy, physical health, and self-determination, among others (Diener, 1984; Hernández Rincón et al., 2022). Another facet that is considered essential for wellbeing is Flourishing.

Flourishing refers to positive traits and states such as purpose, hope, resilience, self-esteem, and positive relationships (Diener et al., 2010). The combination and interaction of the five elements of the PERMA model (Positive Emotions, Engagement, Relationships, Meaning, and Accomplishments) may lead to Flourishing and are key to experience wellbeing (Seligman, 2018). These elements constitute a source of intrinsic reward and thus motivate people to do things and to continue doing them, further allowing the person to achieve optimal psychosocial functioning (Butler and Kern, 2016). In this sense, Flourishing is understood from a humanistic and eudaimonic perspective of wellbeing (Choudhry et al., 2018).

Recent research has provided insights into the importance of Flourishing. For instance, people with high levels of Flourishing tend to have more resourceful coping mechanisms against adverse life events (Prizmić-Larsen et al., 2020). Flourishing also protects against unhealthy lifestyles, risky behaviors, and psychopathological symptoms. As such, research suggests that Flourishing should be considered an essential element in public health interventions (Sofija et al., 2020; Kelly-Hedrick et al., 2022), and be an explicit objective of public policy as it relates to critical elements of social welfare and connectedness with others and the surrounding world (Seaford, 2019).

Although several questionnaires are used to measure Flourishing, this study focused on the Flourishing Scale (FS) (Diener et al., 2010). Originally conceptualized as a unidimensional measure, the FS considers the following constructs: purpose/meaning in life, life satisfaction, optimism, competence, perceived engagement in activities, positive relationships, contributing to others’ happiness, and being respected by others. The scale’s validity, factor structure, and reliability were supported in studies conducted in other countries (Hone et al., 2014; Villieux et al., 2016).

To this day, research on Flourishing in Spanish-speaking populations is sparse, including the validation of measures of Flourishing (Waigel and Lemos, 2023). To our knowledge, the FS has not yet been validated in the Honduran population. It is important that scales are validated for the populations in which they will be used, as cultural and social factors can influence how Flourishing is experienced in different populations. Using a scale that has not been validated for specific countries or cultural groups may inaccurately reflect the experiences of Flourishing of such a population. The current study focuses on the Honduran population, who suffers from high levels of poverty, political instability, and violence (Gindling and Terrell, 2010; Landa-Blanco et al., 2020), that may have a negative impact on their mental health (Knifton and Inglis, 2020; Foell et al., 2021). As such, this population may be in need of interventions aimed at promoting their well-being (including Flourishing) and, in turn, appropriate measures to assess the need for intervention and the effectiveness of such interventions.

Consequently, this study aims to validate the Spanish version of the FS for the Honduran population (Diener and Biswas-Diener, 2009). Based on prior validation studies of the FS, the following hypotheses were tested:

*Hypothesis 1*: The FS has a unidimensional structure with adequate factorial validity. Previous validation studies have found the FS to be a one-factor measurement with adequate fit indices (Diener et al., 2010; Ramírez-Maestre et al., 2017; Choudhry et al., 2018).

*Hypothesis 2*: The FS has adequate internal consistency and test–retest reliability. The original validation of the FS reported a Cronbach’s alpha of 0.87 and a test–retest reliability of 0.71 at a one-month follow-up (Diener et al., 2010).

*Hypothesis 3*: The FS has convergent validity with the scales that assess positive affect and subjective happiness. Validation studies have found a positive correlation between the FS and measurements of self-reported happiness and positive affect (Diener et al., 2010; Hone et al., 2014).

*Hypothesis 4*: The FS has divergent validity with measurements that screen for depression symptoms and negative affect. This hypothesis was supported in the original validation of the FS, in which the scale had significant negative correlations with measurements of negative affect (Diener et al., 2010). More recent validations have found that the FS has an inverse correlation with measurements of depression symptoms (Schotanus-Dijkstra et al., 2016).

*Hypothesis 5*: The FS has configural, metric, scalar, and strict measurement invariance regarding participants’ sex. Prior research validating the FS in the Latin American context, specifically in Colombia, has supported the notion that the scale shows invariance at the configural, metric, and scalar levels, regarding respondents’ sex (Martín-Carbonell et al., 2021).

## 2. Materials and methods

### 2.1. Participants

The primary sample consisted of 422 residents of the Central District of Honduras. Participants’ ages varied between 18 and 69 years (*M* = 28.18; *SD* = 10.58). Most participants were women (*n* = 275; 65.17%), while men accounted for 34.83% (*n* = 147) of the total sample. Participants were included if: (1) 18 years or older, and (2) living in the Central District of Honduras. They were selected through convenience, non-probabilistic sampling; recruitment was made through emails and social media.

A second subsample of 40 students was included solely to determine test–retest reliability, including undergraduate (*n* = 20) and postgraduate (*n* = 20) students. They were selected through convenience, non-probabilistic sampling. Most respondents were women (*n* = 35; 87.50%), while men accounted for 12.50% (*n* = 5) of the subsample. Participants’ age in the second sample ranged between 18 and 47 years (*M* = 25.60; *SD* = 8). All data were collected through online surveys using Google Forms.

### 2.2. Instruments

#### 2.2.1. The Flourishing Scale

The Flourishing Scale (FS) (Diener et al., 2010), that measures Flourishing on a 7-point Likert-type scale, ranging from 1 (strongly agree) to 7 (strongly disagree) is a brief 8-item self-report questionnaire. The item content of the FS relates to different aspects of Flourishing (optimism, positive relationships, competence, meaning in life, being respected by others, and life satisfaction). Summative total scores range between 8 and 56; greater scores indicate a higher self-reported Flourishing. A Spanish version of the FS is available from the authors of the original scale (Diener and Biswas-Diener, 2009); this version was used in the current study.

Previous research made in Spain regarding the Spanish version of the FS, has supported a one-factor solution, which correlated positively with the Satisfaction with Life Scale (*r* = 0.521; *p* > 0.001) and PANAS-Positive Affect subscale (*r* = 0.422; *p* > 0.001), and inversely related with PANAS-Negative Affect subscale (*r* = −0.270; *p* > 0.001). The mentioned research also concluded that the Spanish version of the FS had adequate internal consistency (*α* = 0.846) and temporal reliability (*r* = 0.749; *p* > 0.001) (Checa et al., 2018).

#### 2.2.2. The subjective happiness scale

The Subjective Happiness Scale (SHS) (Lyubomirsky and Lepper, 1999), is a 4-item Likert-type scale, ranging from 1 (totally disagree) to 7 (totally agree). Total summative scores range from 4 (low subjective happiness) to 28 (high subjective happiness). The original validation of the SHS reports a single-factor structure with adequate reliability (*α* = 0.86) and convergent validity with other happiness measures (*r* = 0.62, *p* < 0.01) (Lyubomirsky and Lepper, 1999). Based on the data collected in the current study, the SHS has an adequate internal consistency, *ω* = 0.75, *CI* 95% [0.71, 0.78]. A CFA yielded the following fit indices for the single-factor model: *χ^2^*(2) = 1.162; *p* = 0.559; CFI = 0.999, GFI = 0.999; TLI = 0.999; RMSEA = 0.001; SRMR = 0.008. The SHS was included to test Hypothesis 3.

#### 2.2.3. The Positive and Negative Affect Schedule

The Positive and Negative Affect Schedule (PANAS) is a widely used questionnaire that evaluates positive and negative affect (Díaz-García et al., 2020). It consists of 20 items, of which 10 measure positive affect (for example: interested, excited, strong) and the other 10 measure negative affect (for example: distressed, upset, scared), as experienced during the last week. Each item is rated on a 5-point Likert-type scale, ranging from 1 (very slightly or not at all) to 5 (extremely). Higher scores indicate a higher affect intensity. The original validation study of the PANAS reported adequate internal consistency coefficients for the Positive Affect subscale (*α* = 0.88) and the Negative Affect subscale (*α* = 0.87) (Watson et al., 1988). Based on the current study, the PANAS achieved an overall high reliability, *ω* = 0.85, *CI* 95% [0.83, 0.87]. A CFA yielded the following fit indices for the two-factor model: *χ^2^*(169) = 674.864; *p* < 0.001; CFI = 0.873; GFI = 0.971; TLI = 0.857; RMSEA = 0.084; SRMR = 0.070. The PANAS-Positive Affect subscale was included to test Hypothesis 3, while the PANAS-Negative Affect subscale was necessary to test Hypothesis 4.

#### 2.2.4. Patient Health Questionnaire-9

The Patient Health Questionnaire-9 (PHQ-9) is a 9-item questionnaire used to screen for symptoms of depression (Kroenke and Spitzer, 2002). The scale asks participants to rate the presence of specific symptoms, as experienced during the last 2 weeks, on a 4-point Likert-type scale ranging from 0 (not at all) to 3 (nearly every day). Total summative scores range between 0 (low symptom prevalence) to 27 (high symptom prevalence). A validation of the PHQ-9 in a Latin American country -Peru- has supported a unidimensional model and high reliability (*ω* = 0.87) (Villarreal-Zegarra et al., 2019). Prior studies made in the Honduran population have found that the PHQ-9 has adequate reliability (*α* = 0.80) (Martínez-Martínez et al., 2021). Data from the current study further supports such internal consistency, *ω* = 0.90, *CI* 95% [0.88, 0.91]. A CFA yielded the following fit indices for the single-factor model: *χ^2^*(27) = 71.680; *p* < 0.001; CFI = 0.974; GFI = 0.981; TLI = 0.965; RMSEA = 0.063; SRMR = 0.028. The PHQ-9 was included to test Hypothesis 4.

### 2.3. Ethical considerations and procedures

The research was done in accordance with the principles stated in the Declaration of Helsinki and the Code of Ethics of the Honduran Psychologist. This study was approved by the Research Ethics Committee of the Faculty of Social Sciences (UNAH) under registration CEIFCS-P1-2023. The informed consent form was presented to all potential participants recruited via email and social media. After reading the document, respondents were presented with closed (yes/no) questions that assessed whether: (1) they agreed to the informed consent, (2) they gave permission to collect their data, and (3) they participated voluntarily. Agreeing to the three questions was mandatory to start the online survey. The survey did not collect personal information to ensure anonymity. Data were collected from February to March of 2023. Test–retest follow-up was set at the one-month mark, following the original validation of the FS (Diener et al., 2010).

### 2.4. Data analyses

All data was collected online through Google Forms. All answers were mandatory; therefore, the dataset does not contain missing data. Later, where applicable, negatively worded items were reverse scored, and summative totals were obtained for each scale. The statistical analyses were run on JASP (JASP Team, 2020). Descriptive measures for the FS include mean scores (*M*), standard deviations (*SD*), skewness, and kurtosis. A one-sample *z*-test was used to compare the mean scores of the FS in the Honduran sample with those of the original validation study in the American population (Diener et al., 2010). FS mean score comparisons by sex were made using Student’s *t*-test for independent groups; effect size was estimated through Cohen’s *d*. All testing was done at a 95% confidence level.

Later, an Exploratory Factor Analysis (EFA) and a Confirmatory Factor Analysis (CFA) were used to assess the scale’s factorial validity (Hypothesis 1); Bartlett’s Test of Sphericity was also reported. The following fit indices for EFA and CFA were reported and analyzed using the following acceptability thresholds (Parry, 2017): Root Mean Square Error of Approximation (RMSEA) <0.08, Comparative Fit Index (CFI) > 0.90, and Tucker-Lewis Index (TLI) >0.95. A general CFA assessment was made with no-group comparisons. Goodness of Fit (GFI) was also reported for the CFA, with scores >0.95 suggesting adequate fit (Parry, 2017).

Test–retest reliability was determined through a dependent sample *t*-test and Spearman’s rho at a one-month follow-up (Hypothesis 2). The internal consistency of the FS was estimated using McDonald’ *ω*; scores higher than 0.75 are considered adequately reliable (Coolican, 2019). Then, convergent validity was investigated through Pearson’s *r* correlation coefficients between the FS, the Subjective Happiness Scale, and the Positive Affect subscale of the PANAS (Hypothesis 3). On the other hand, divergent validity was obtained by correlating the FS with the Patient Health Questionnaire-9 and the Negative Affect subscale of the PANAS (Hypothesis 4).

Finally, invariance measurements were obtained for multi-group comparisons based on the respondents’ sex (Hypothesis 5). A four-stage approach was used, consisting of: (1) configural invariance (no equality constraints), (2) metric invariance (loading equality constraints), (3) scalar invariance (loadings and intercept equality constraints), and (4) strict invariance (loadings, intercept, residual and residual covariance equality restraints). Changes (Δ) in CFI, Standardized Root Mean Square Residual (SRMR), and RMSEA scores were evaluated to determine measurement invariance. Using a similar methodology as recent validations of the FS (Romano et al., 2020), the following thresholds were identified: ΔCFI ≤ −0.010, ΔSRMR ≥0.030, or ΔRMSEA ≥0.015. At least two coefficient changes had to fit the thresholds to consider measurement invariance.

## 3. Results

### 3.1. Description of the Flourishing Scale items

The overall FS score was 46.65 (*SD* = 8.05); this is higher than the score in the original validation of the FS (*M* = 44.97; *SD* = 6.56), as Diener et al. (2010) reported. A one-sample *z*-test determined this difference was statistically significant (*p* > 0.01). Total FS scores had a skewness of −1.34 and a kurtosis of 2.06. Individual items’ mean scores ranged between 5.47 (*SD* = 1.43; Item 2) and 6.20 (*SD* = 1.22; Item 5). Overall, the total FS score did not vary significantly between men (*M* = 47.37; *SD* = 7.50) and women (*M* = 46.26; *SD* = 8.32), *p* = 0.18. However, men scored significantly higher in Item 1 and Item 7, see Table 1.

**Table 1:** Descriptive Flourishing Scale scores.

Item	Total			Men			Women			*t*	*d*	*p*
	Mean (*SD*)	Skewness	Kurtosis	Mean (*SD*)	Skewness	Kurtosis	Mean (*SD*)	Skewness	Kurtosis			
Item 1	5.74 (1.45)	−1.20	1.01	5.96 (1.37)	−1.45	1.65	5.62 (1.48)	−1.10	0.83	2.29	0.23	**0.02**
Item 2	5.47 (1.43)	−0.90	0.22	5.52 (1.38)	−0.75	−0.19	5.44 (1.46)	−0.96	0.36	0.50	0.05	0.62
Item 3	5.84 (1.41)	−1.32	1.35	5.90 (1.28)	−1.06	0.41	5.80 (1.48)	−1.39	1.51	0.65	0.07	0.51
Item 4	5.89 (1.20)	−1.22	1.74	5.86 (1.19)	−1.09	1.40	5.91 (1.20)	−1.29	1.97	−0.39	−0.04	0.69
Item 5	6.20 (1.22)	−2.23	5.86	6.18 (1.24)	−2.13	5.45	6.21 (1.21)	−2.30	6.23	−0.19	−0.02	0.85
Item 6	5.87 (1.26)	−1.37	2.19	5.99 (1.18)	−1.36	2.04	5.80 (1.29)	−1.36	2.22	1.48	0.15	0.14
Item 7	5.74 (1.60)	−1.33	1.01	6.03 (1.31)	−1.71	3.22	5.59 (1.72)	−1.14	0.31	2.70	0.28	**0.01**
Item 8	5.90 (1.29)	−1.50	2.46	5.94 (1.25)	−1.63	3.21	5.88 (1.32)	−1.44	2.18	0.42	0.04	0.68
Total	46.65 (8.05)	−1.34	2.06	47.37 (7.50)	−1.49	2.95	46.26 (8.32)	−1.27	1.73	1.36	0.14	0.18

The sample consisted of 147 men and 275 women; df = 420. Significant *p*-values (*p* < 0.05) are marked in bold. Standard deviation = SD; *t* = Student’s *t*-test; *d* = Cohen’s d effect size estimator; *p* = *p*-value.

### 3.2. Factorial validity

Exploratory Factor Analysis (EFA) and Confirmatory Factor Analysis (CFA) were performed to assess the factor structure of the FS. Bartlett’s Test of Sphericity yielded a statistically significant coefficient (*X*^2^ = 1536.75, *df* = 28, *p* < 0.001), suggesting the correlation matrix is suitable for factor analysis. EFA analysis suggests a unidimensional structure, see Table 2. All items show a high factor loading (>0.50), with a maximum likelihood method General MSA of 0.90. The rotated solution achieved a proportion variance of 0.49; *X^2^*(20) = 92.63; *p* < 0.001. According to the EFA, the unidimensional model achieved adequate fit indices: CFI = 0.95; TLI = 0.93; SRMR = 0.042, RMSEA = 0.09. Additionally, the global CFA results suggest further evidence in favor of the unidimensional factor structure of the FS (CFI = 0.95; TLI = 0.93; RMSEA = 0.09; GFI = 0.99); this supports Hypothesis 1.

**Table 2:** EFA structure of the Flourishing Scale.

Item	Factor 1	Uniqueness	MSA
Item 3	0.81	0.34	0.91
Item 1	0.81	0.34	0.90
Item 7	0.78	0.39	0.88
Item 6	0.78	0.40	0.92
Item 5	0.70	0.51	0.92
Item 8	0.58	0.66	0.92
Item 2	0.56	0.69	0.91
Item 4	0.52	0.73	0.88

The model was built using a maximum likelihood estimation method and an oblique-oblimin rotation. MSA, Kaiser’s Measure of Sampling Adequacy.

### 3.3. Reliability of the Flourishing Scale

The FS achieved a high internal consistency with McDonald’s *ω* = 0.89, 95% *CI* [0.86, 0.91]. The average inter-item correlation was 0.48, 95% *CI* [0.43, 0.53]. Using Student’s *t*-test for paired samples, results indicate that none of the FS items varied significantly between baseline and post-test, see Table 3. Additionally, Spearman’s rho was used to correlate test–retest scores; this yielded a statistically significant correlation coefficient of 0.66 (*p* < 0.001). These findings suggest the FS has adequate temporal reliability at the one-month follow-up, supporting Hypothesis 2.

**Table 3:** Test–retest reliability coefficients of the Flourishing Scale.

Test–retest	*t*	*p*	Cohen’s *d*
Item 1	−1.58	0.12	−0.25
Item 2	−0.63	0.53	−0.10
Item 3	−1.33	0.19	−0.21
Item 4	−1.64	0.11	−0.26
Item 5	−1.42	0.16	−0.22
Item 6	−1.12	0.27	−0.18
Item 7	−0.91	0.37	−0.14
Item 8	0.40	0.69	0.06
Total scale	−1.34	0.19	−0.21

Student’s *t*-test for paired samples; df = 39. *t* = Student’s *t*-test; *d* = Cohen’s d effect size estimator; *p* = *p*-value.

### 3.4. Convergent and divergent validity

Using Pearson’s *r* coefficient, the FS had adequate convergent validity with the Subjective Happiness Scale (*r* = 0.70; *p* < 0.001) and the PANAS-Positive Affect Subscale (*r* = 0.70; *p* < 0.001), thus supporting Hypothesis 3. Coherently, it correlates inversely with the PANAS-Negative Affect Subscale (*r* = −0.34; *p* < 0.001) and the PHQ-9 (*r* = −0.51; *p* < 0.001), supporting Hypothesis 4. Altogether, these findings provide support in favor of the validity of the FS.

### 3.5. Measurement invariance

Based on the differences in CFI, SRMR, and RMSEA, the results suggest that the FS has metric, scalar, and strict measurement invariance with regard to the respondents’ sex (Hypothesis 5), see Table 4.

**Table 4:** Measurement invariance of the Flourishing Scale.

Invariance	*χ^2^* (*df*)	CFI	SRMR	RMSEA	Δ *χ^2^* (*df*)	Δ CFI	Δ SRMR	Δ RMSEA
Configural	128.511 (40)	0.943	0.044	0.102	–	–	–	–
Metric	141.483 (47)	0.939	0.060	0.098	12.972 (7)	−0.004	0.016	−0.004
Scalar	158.090 (54)	0.932	0.064	0.096	16.607 (7)	−0.007	0.004	−0.002
Strict	169.845 (62)	0.93	0.068	0.091	11.755 (8)	−0.002	0.004	−0.005

χ^2^, Chi-square; df, degrees of freedom; CFI, Comparative Fit Index; SRMR, 0.028; RMSEA, Standardized Root Mean Square Residual; Δ, difference.

## 4. Discussion

The results support the unidimensionality of the Spanish version of the FS. The one-factor model has also been supported in other studies within the Latin American context (Da Fonseca et al., 2015). The FS also has adequate test–retest reliability at the one-month follow-up, with a *rho* of 0.66, constituting a large effect size (Cohen, 1992). Additionally, the FS achieved a high internal consistency (McDonald’s *ω* = 0.89).

Regarding validity, scores on the FS are positively related to scores on the SHS and the PANAS-Positive Affect subscale. This is in line with the theoretical dynamic between such variables, as happiness and positive feelings are core components of Flourishing (Weele and Tyler, 2017). Additional support for the validity of the FS was found when determining its inverse relationship with scores on the PHQ-9 and PANAS-Negative Affect subscale. Previous research has also concluded that Flourishing is a protective factor against poor mental health outcomes (Burns et al., 2022). The data supports the metric, scalar, and strict measurement invariance of the Spanish version of the FS concerning the respondents’ sex.

Measuring Flourishing is helpful for several reasons. Firstly, it allows researchers and policymakers to understand the factors contributing to wellbeing and happiness among individuals and communities (Weele and Tyler, 2017). Through an evidence-based approach, this understanding can be used to develop policies and programs that are more effective in promoting human development and reducing poverty and inequality. Such an approach may inform strategic decisions for developing programs and public policies aimed at preventive aspects of mental health promotion (Trudel-Fitzgerald et al., 2019).

Secondly, measuring Flourishing can help identify populations at risk of poor wellbeing and needing intervention (Roche et al., 2019). The effectiveness of existing policies and programs can be evaluated through measurements of Flourishing. This information can then be used to improve the design and implementation of future interventions based on population needs. Flourishing measurements can provide a benchmark for the progress of society and the government’s efforts to improve the wellbeing of its citizens. It is a way to track the progress and adjust the policies accordingly, ensuring that society is moving toward social development (Landa-Blanco, 2021). Measuring Flourishing can also provide valuable insights into the impact of certain events, such as natural disasters, economic downturns, or pandemics, on the wellbeing of individuals and communities. Considering many of these events are unpredictable, having a Flourishing baseline readily available through universal screenings may be useful; as measuring Flourishing before and after these events, researchers and policymakers can understand the event’s impact and develop interventions to mitigate any adverse effects on wellbeing.

However, the present study has limitations that must be considered. Firstly, the non-probabilistic sampling selection limits the generalization of the results. Future studies should aim to include more representative samples from Honduras and other global populations. Secondly, data were collected online. Therefore, people without access to the internet or technological devices, such as computers or mobile phones, could not participate in the survey. Thirdly, no linguistic validation of the FS was done; this could have provided insights into respondents’ interpretation of individual items. Fourthly, replications and further studies are needed to understand the FS functioning in low-and-middle-income countries, as the overall research production of the global South is still limited (Albanna et al., 2021); this has led to a significant gap in the literature regarding Flourishing in Spanish-speaking countries (Waigel and Lemos, 2023).

In conclusion, the current data support the one-factor structure of the Spanish version of the FS in the Honduran population. The scale also had high internal consistency and temporal reliability at the one-month follow-up. Convergent and divergent validity has been established, as well as metric, scalar, and strict measurement invariance concerning the respondents’ sex. Validating the FS in specific populations, like Honduras, is essential to ensure the scale is appropriate and relevant for the contexts and cultures in which they will be used. It can also be used to better understand Flourishing in the Honduran and other global populations, which, in turn, can also facilitate the development of interventions and programs that promote wellbeing.

## Data availability statement

The raw data supporting the conclusions of this article will be made available by the authors, without undue reservation.

## Ethics statement

The studies involving human participants were reviewed and approved by the Comité de Ética en Investigación de la Facultad de Ciencias Sociales (UNAH). The patients/participants provided their written informed consent to participate in this study.

## Author contributions

ML-B: conceptualization, methodology, validation, formal analysis, investigation, supervision, project administration, and writing—original draft. AC-R: conceptualization, methodology, validation, investigation, and writing—original draft. GV, YR, and YE: conceptualization, methodology, validation, and investigation. All authors involved approved the final version of the manuscript to be published.

## Conflict of interest

The authors declare that the research was conducted in the absence of any commercial or financial relationships that could be construed as a potential conflict of interest.

## Publisher’s note

All claims expressed in this article are solely those of the authors and do not necessarily represent those of their affiliated organizations, or those of the publisher, the editors and the reviewers. Any product that may be evaluated in this article, or claim that may be made by its manufacturer, is not guaranteed or endorsed by the publisher.
